# Evaluating procollagen type III N-terminal propeptide (P3NP) as a biomarker of sarcopenia in women with type 2 diabetes: a cross-sectional study

**DOI:** 10.20945/2359-4292-2026-0118

**Published:** 2026-10-11

**Authors:** Thyago Bruno de Lira dos Santos, Giselle Rabelo Maciel, Francisco Bandeira

**Affiliations:** 1 Programa de Pós-Graduação em Ciências da Saúde, Universidade de Pernambuco, Recife, PE, Brasil; 2 Divisão de Endocrinologia e Diabetes, Hospital Agamenon Magalhães, Recife, PE, Brasil

**Keywords:** Sarcopenia, diabetes mellitus, type 2, biomarkers, procollagen type III

## Abstract

**Objective:**

To determine whether circulating procollagen type III N-terminal propeptide (P3NP) improves the diagnosis of dual-energy X-ray absorptiometry (DXA)-defined sarcopenia in women with type 2 diabetes mellitus (T2DM).

**Subjects and methods:**

This cross-sectional study enrolled 99 women with T2DM who underwent standardized clinical assessment, handgrip strength testing, and whole-body DXA. Sarcopenia was defined using a DXA-derived fat-free mass index. Multivariable logistic regression models included age, body mass index, glycated hemoglobin (HbA1c), and estimated glomerular filtration rate, with and without log-transformed, z-standardized P3NP. Discriminatory performance was quantified by the area under the curve (AUC) with bootstrap 95% confidence intervals; sensitivity analyses excluded participants with an estimated glomerular filtration rate below 60.

**Results:**

P3NP showed weak bivariate correlations with age (p = -0.210) and the Short Physical Performance Battery (p = 0.225). When incorporated into the clinical model, P3NP did not yield a clinically significant improvement (ΔAUC = 0.007). As a sole predictor, P3NP had poor discrimination (AUC 0.53) and was not independently associated with DXA-defined sarcopenia (odds ratio per 1-standard deviation, *p* > 0.05). Results were consistent after excluding participants with reduced kidney function and across interaction analyses; model calibration was adequate. P3NP absolute values were elevated, with 80.8% above the high threshold (>31 µg/L), and clinical/functional profiles were broadly similar P3NP strata.

**Conclusion:**

In women with T2DM, circulating P3NP was not an independent discriminator of DXA-defined sarcopenia and provided no clinically meaningful advantage over standard clinical predictors. Routine screening should continue to rely on assessments of muscle strength, function, and DXA-based body composition, while P3NP may be considered an adjunct marker of extracellular matrix remodeling.

## INTRODUCTION

Sarcopenia is a progressive, generalized loss of skeletal muscle mass and function, with major consequences for disability, frailty, and mortality (^1^,^2^). Its underlying biology is multifactorial, involving hormonal changes, neuromuscular junction degeneration, chronic low-grade inflammation, and dysregulated remodeling of the extracellular matrix. Early recognition is critical because declines in strength and performance typically precede disability and are more e to early intervention (^1^,^3^,^4^,^5^,^6^).

Women with type 2 diabetes mellitus (T2DM) a high-risk group. In this population, biomarker-phenotype relationships may differ and should be interpreted adiposity, renal function, and treatment patterns. Evidence focused on women with T2DM and dual-energy X-ray absorptiometry (DXA)-based definitions remains limited, and common comorbidities can blunt muscle-specific signals (^7^,^8^,^9^,^10^). In clinical practice, blood biomarkers are best used as adjuncts to standardized phenotyping with DXA assessment, muscle strength testing, and evaluation of physical performance (^11^,^12^).

Procollagen type III N-terminal propeptide (P3NP), also PIIINP, reflects extracellular matrix turnover; however, it is not muscle-specific and may be elevated in the context of cardiac, hepatic, or adipose tissue remodeling. In diabetes, metabolic alterations may further modulate its signal (^13^,^14^,^15^). Studies investigating the association between P3NP and sarcopenia have shown mixed results across studies employing differing definitions, body composition methods, assay platforms, and population characteristics, including diabetes prevalence, adiposity, and renal function (^13^,^16^,^17^,^18^,^19^,^20^,^21^,^22^). Given the multifactorial nature of sarcopenia, recent research has shifted towards the use of composite biomarker panels and has emphasized the need for assay harmonization, while maintaining biomarkers as complementary to standardized phenotypes (^11^,^12^,^23^,^24^,^25^,^26^).

Given this context, this study aimed to determine whether circulating P3NP improves discrimination of DXA-defined sarcopenia beyond a parsimonious clinical model including age, body mass index (BMI), glycated hemoglobin (HbA1c), and estimated glomerular filtration rate (eGFR) in women with T2DM.

## SUBJECTS AND METHODS

### Study design and setting

A single-center, observational, cross-sectional study was conducted at the Endocrinology and Diabetes Unit of Hospital Agamenon Magalhães (Recife, Brazil) between July 2024 and May 2025. All evaluations (clinical assessment, functional testing, venous blood sampling, and DXA) were performed during a single outpatient visit. Reporting followed the Strengthening the Reporting of Observational Studies in Epidemiology (STROBE) guideline for cross-sectional studies.

### Participants

Consecutive adult women (≥18 years) with T2DM receiving routine care at the clinic were enrolled. Major exclusion criteria included significant cardiovascular disease, advanced chronic kidney disease (eGFR <30 mL/min/1.73 m^2^), severe mobility-limiting orthopedic conditions, proliferative retinopathy, disabling peripheral neuropathy, New York Heart Association class III-IV heart failure, moderate-to-severe hepatic dysfunction, uncontrolled thyroid disease, and active malignancy.

The study was conducted in accordance with the Declaration of Helsinki and Brazilian Resolution 466/12, and received approval from the hospital's research ethics committee (CAAE no. 49119021.5.0000.5197; approval no. 4.915.754). All participants provided written informed consent.

### Sample size and power

A consecutive convenience sample of 99 adult women with T2DM was recruited, determined by feasibility during the study period (July 2024 to May 2025). No formal a priori sample size calculation was performed. Consistent with STROBE recommendations, results are presented with an emphasis on estimate precision (95% confidence intervals [CIs]) and minimum detectable effects rather than *post-hoc* power analyses.

For the primary logistic models (DXA-defined sar-copenia as the outcome), there were 67 events among 99 participants, resulting in an events-per-variable (EPV) ratio of approximately 13 for the prespecified parsimonious clinical model (age, BMI, HbA1c, eGFR, plus log-P3NP), and about 11 when including a single interaction term. These EPV values are compatible with stable estimation in the primary analyses. In contrast, stratified models showed lower EPV: about 9 in the high HbA1c stratum (n = 56; 37 events) and about 7.5 in the low HbA1c stratum (n = 43; 30 events), indicating reduced power and increased risk of over-fitting; thus, findings from these exploratory strata should be interpreted with caution.

For bivariate correlations (two prespecified: P3NP with age; P3NP with Short Physical Performance Battery [SPPB]), with n = 99 and α = 0.05 (two-sided), the minimum detectable effect for an absolute Spearman correlation was roughly *p* ≈ 0.28-0.30, and a correlation near *p* ≈ 0.20 would have 95% CI half-width of approximately ±0.19, reflecting modest precision.

For between-group comparisons by sarcopenia status (67 vs. 32), α = 0.05 provided roughly 80% power only for moderate standardized differences (Cohen's d ≈ 0.6); smaller differences were likely underpowered. Analyses of categorical distributions (e.g., P3NP categories across sarcopenia status) were further limited by small cell counts (e.g., the <14 µg/L group had n = 4), constraining the ability to detect small deviations from the null and potentially affecting test validity.

No interim analyses or stopping rules were applied. Inferential analyses used two-sided p-values (α = 0.05). For the two prespecified correlations, both raw and Holm-adjusted *p*-values are reported. Given the limited sample for detecting small improvements in discrimination, ΔAUC estimates are presented with bootstrap confidence intervals and interpreted alongside cross-validated area under the curve (AUCs) to avoid overoptimistic conclusions.

### Measurements and procedures

After obtaining written informed consent, all evaluations were completed during a single outpatient visit by trained personnel. Laboratory staff were blinded to clinical and sarcopenia data. A standardized case-report form was used to collect sociodemographic data, diabetes duration, comorbidities, current medications, and lifestyle factors (e.g., physical activity, smoking, alcohol consumption). Anthropometric measurements and vital signs included weight, height, BMI (kg/m^2^), waist circumference, blood pressure, heart rate, and peripheral oxygen saturation. Peripheral neuropathy was assessed with a 10 g Semmes-Weinstein monofilament applied to both feet. Handgrip strength was measured with a hydraulic dynamometer (EH101, CAMRY, China) in the dominant hand while seated (shoulder adducted, elbow at 90°, forearm neutral, wrist slightly extended); three trials were performed with 1-minute rest intervals, and the maximum value (kg) was retained. Physical performance was assessed with the SPPB (score 0-12) by a trained assessor. Data abstracted from electronic medical records included fasting plasma glucose, HbA1c, lipid profile (total cholesterol, LDL, HDL, triglycerides), creatinine (used for eGFR by CKD-EPI), calcium, and vitamin D.

Whole-body DXA scans were performed on a GE Lunar Prodigy system following manufacturer guidelines. Fat-free mass (FFM) and appendicular lean mass (ALM) were obtained from the scans. Fat-free mass index (FFMI) was calculated as FFM/height^2^, and ALM/height^2^ (Baumgartner index) and ALM/BMI (Foundation for the National Institutes of Health [FNIH] index) were also derived. Body composition analyses were performed by a single reader blinded to P3NP results. For biomarker analysis, venous blood was collected in serum gel tubes, allowed to clot, cen-trifuged at 3,000 rpm for 5 min, with serum aliquoted and stored at -30 °C, underwent a single freeze-thaw cycle, and analyzed in one batch. P3NP was measured in µg/L by chemiluminescent immunoassay (MAGLUMI PIIIP N-P kit on the MAGLUMI X8 analyzer, Snibe, China) according to the manufacturer's instructions and routine internal quality-control procedures. Laboratory personnel performing P3NP assays were blinded to all clinical data and sarcopenia status; samples were labeled only with anonymized study IDs.

### Definitions

Sarcopenia was staged according to the 2019 EWGSOP2 criteria, integrating muscle strength, muscle quantity, and physical performance (^27^). Low strength was defined as handgrip <16 kg in women, and poor performance as SPPB ≤8. Low muscle quantity was determined using DXA-derived FFMI with prespecified BMI-specific thresholds for women to account for adiposity: FFMI ≤14.6 kg/m^2^ (BMI ≤18.5), ≤15.1 (18.5-20.0), ≤16.8 (20.0-25.0), ≤17.5 (25.0-27.3), and ≤18.2 (>27.3, including ≥30). Although sarcopenia was conceptually aligned with EWGSOP2 criteria, the primary outcome in this study was defined by DXA-derived FFMI to specifically evaluate the discriminatory performance of P3NP for muscle mass, independent of functional components.

P3NP results were interpreted against MAGLUMI-based reference intervals as reported by Larsen and cols. (^22^) for healthy adults using the same assay/platform. Given the study age range (41-86 years), P3NP values were pre-categorized as low (<14 µg/L), normal (14-31 µg/L), and high (>31 µg/L) for descriptive analyses; for inferential analyses, P3NP was log-transformed, z-standardized, and treated as a continuous variable.

Baseline clinical definitions followed standard criteria: hypertension was defined as current use of any an-tihypertensive medication; smoking as current use or cessation within the past 10 years (others were considered non-smokers/ex-smokers ≥10 years); alcohol use as current consumption or cessation within 3 months (otherwise, non-use/ex-use ≥3 months); menopause as ≥l2 months of amenorrhea; and physical activity level was classified according to the International Physical Activity Questionnaire (IPAQ), Short Form (Brazilian version), following official scoring guidelines.

### Statistical methods

Continuous variables were summarized as mean ± standard deviation (SD) or median (interquartile range), and categorical variables as n (%). Distributions were assessed using histograms and Q-Q plots; normality was evaluated with the Shapiro-Wilk test, and homoscedasticity with Levene's test. Between-group comparisons (DXA-FFMI sarcopenia: yes/no) used two-sided Student's *t* (Welch's when variances were unequal) or Mann-Whitney U; effect sizes (95% CIs) were Hedges' g/Cohen's d for *t*-tests and rank-biserial r plus the Hodges-Lehmann median difference for Mann-Whitney. Categorical contrasts used Fisher's exact (χ^2^ avoided under sparsity). For P3NP categories (<14, 14-31, >31 µg/1), continuous outcomes used Kruskal-Wallis (ε^2^) with Dunn-Holm *post hoc* testing if the omnibus test was significant; categorical strata (e.g., sarcopenia stages, comorbidities, IPAQ, SPPB) were evaluated with Fisher's exact test, reporting exact odds ratio (ORs; 95% CIs) and Cramér's V. The two prespecified correlations (P3NP-age; P3NP-SPPB) were assessed using two-tailed Spearman's *p* with Holm adjustment and Fisher's z CIs. Exploratory scatterplots with LOESS smoothers were examined to assess potential nonlinearity.

Sarcopenia was modeled using multivariable logistic regression: a prespecified clinical model (age, BMI, HbA1c, eGFR; all z-standardized), and the same model plus log-transformed, z-standardized P3NP (the primary exposure). P3NP was also summarized per 10 µg/L. Linearity in the logit was examined via component-plus-residual plots with LOESS and by comparing models that included quadratic terms; potential non-linearity for HbA1c was explored with restricted cubic splines (degrees of freedom [df] = 4). Discriminative ability was evaluated using the ROC AUC with bootstrap-derived 95% percentile CIs (B = 2,000); ΔAUC was estimated with bootstrap CIs and corroborated using DeLong's method as a sensitivity analysis. Calibration metrics included the Hosmer-Lemeshow test, Brier score, calibration intercept/slope, and decile-based calibration plots.

Effect modification was assessed a priori for P3NP × BMI (centered z-scores) and explored for P3NP × HbA1c (binary <7.4%/≥7.4% and continuous splines), with marginal effects reported at BMI quartiles and at HbA1c values of 6.5%, 7.4%, and 8.5%, holding other covariates at sample means. The stratification of HbA1c into <7.4% and ≥7.4% was based on epidemiological evidence indicating increased all-cause mortality above this threshold in individuals with diabetes. Large population-based, penalized spline models have identified an inflection point around 7.37%, beyond which mortality risk increases significantly, with the nadir near 6.5%; therefore, 7.4% was used as a pragmatic cutoff for this transition. This threshold is not a guideline-based therapeutic target but is instead based on nonlinear mortality modeling showing a U-shaped association between HbA1c and survival (^28^). Internal validation employed bootstrap optimism correction (B = 200) and 5-fold cross-validation for AUC, both overall and stratified by HbA1c. All tests were two-sided (a = 0.05), with multiplicity correction limited to the two prespecified correlations (Holm). Analyses were performed in jamovi using the Rj Editor (R).

### Treatment of missing data

There were no missing data in primary variables or key covariates; all analyses used complete cases.

## RESULTS

From July 2024 to May 2025, adults with T2DM attending the Endocrinology and Diabetes Clinic at Hospital Agamenon Magalhães were screened. Of l2l individuals assessed, 22 were excluded (ineligible, declined participation, or hemolyzed samples), resulting in 99 participants included in the analysis **(Supplementary Figure S1).**

Across prespecified and exploratory analyses, inclusion of P3NP in the clinical model did not yield clinically meaningful gains in discrimination (ΔAUC ≈ 0.007). As shown in **Table 1,** women with sarcopenia by DXA-derived FFMI (n = 67) were older and had longer diabetes duration than those without sarcopenia. Significant differences were observed in body composition, with the sarcopenia group exhibiting substantially lower BMI, waist circumference, and DXA-derived muscle mass indices (all *p* < 0.05). Handgrip strength was lower in the sarcopenia group; however, SPPB scores were comparable between groups. Metabolic and renal biomarkers, including glycemic control and kidney function, did not significantly differ between groups.

**Table 1:** Baseline clinical and laboratory characteristics of participants according to sarcopenia status defined by FFMI-DXA

**Variable**	**Sarcopenia by FFMI-DXA**		***p*-Value**
	**Yes (n = 67)**	**No (n = 32)**	
Clinical variables			
Age, years	61.35 ± 8.79	56.3 ± 9.12	0.010*
Diabetes duration, years	12.34 ± 10.52	7.68 ± 6.09	0.040#
BMI, kg/m^2^	28.23 ± 3.62	36.86 ± 7.43	<0.001#
Waist circumference, cm	96.72 ± 8.86	112.87 ± 13.43	<0.001#
Handgrip strength, kg	23.48 ± 5.10	25.89 ± 5.18	0.031*>
SPPB score, 0-12	11.13 ± 1.53	11.53 ± 0.91	0.376#
Baumgartner index, kg/m^2^	6.94 ± 0.81	8.88 ± 1.11	<0.001^#^
FNIH index	0.60 ± 0.07	0.62 ± 0.16	0.982#
Laboratory variables			
HbA1c, %	8.01 ± 2.24	7.95 ± 1.86	0.943#
Fasting glucose, mg/dL	144.94 ± 62.76	150.56 ± 61.46	0.334#
eGFR (CKD-EPI), mL/min/1.73 m^2^	91.45 ± 21.23	99.50 ± 12.98	0.190#
LDL cholesterol, mg/dL	102.20 ± 45.81	112.08 ± 36.20	0.150#
HDL cholesterol, mg/dL	56.90 ± 16.88	54.95 ± 16.25	0.659#
Triglycerides, mg/dL	155.48 ± 107.08	164.49 ± 74.52	0.194#
Total cholesterol, mg/dL	190.09 ± 54.38	200.47 ± 47.29	0.263#
Vitamin D, ng/mL	31.45 ± 9.31	30.47 ± 10.54	0.434#
Calcium, mg/dL	9.47 ± 1.53	10.49 ± 4.29	0.899#
P3NP, µg/L	76.82 ± 50.20	88.57 ± 66.99	0.614#
log_P3NP	1.79 ± 0.30	1.79 ± 0.45	0.614#

Values are presented as mean ± SD. BMI: body mass index; CKD-EPI: chronic kidney disease epidemiology collaboration; eGFR: estimated glomerular filtration rate; FFMI-DXA: fat-free mass index by DXA; FNIH: DXA-derived appendicular lean mass/BMI; HbA1c: glycated hemoglobin; LDL/HDL: low/high-density lipoprotein; P3NP: procollagen type III N-terminal propeptide; SPPB: Short Physical Performance Battery; WC: waist circumference. *Student's t-test. ^#^Mann-Whitney U test.

Absolute P3NP levels were generally elevated relative to platform-specific reference intervals: 80.8% of participants were in the high category (>31 µg/L), l5.2% in the normal range (l4-3l µg/L), and 4.0% in the low range (<l4 µg/L). Within these categories **(Table 2),** clinical, functional, and body composition measures were similar: age (*p* = 0.299), diabetes duration (*p* = 0.375), BMI (*p* = 0.886), waist circumference (*p* = 0.956), handgrip strength (*p* = 0.362), SPPB score (*p* = 0.l95), FFMI-DXA (*p* = 0.690), Baumgartner index (*p* = 0.730), and FNIH (*p* = 0.l03). Laboratory profiles were also comparable across P3NP groups, including HbA1c (*p* = 0.683), fasting glucose (*p* = 0.459), eGFR (*p* = 0.228), LDL (*p* = 0.956), HDL (*p* = 0.832), triglycerides (*p* = 0.954), total cholesterol (*p* = 0.859), vitamin D (*p* = 0.968), and calcium (*p* = 0.54l).

**Table 2:** Clinical and laboratory characteristics across P3NP categories

**Variable**	**P3NP levels (µg/L)**			*p*-Value*
	**< 14**	**14-31**	**> 31**	
	**Low (n = 4)**	**Normal (n = 15)**	**High (n = 80)**	
Clinical variables				
Age, years	61.00 (2.00)	61.00 (8.50)	58.50 (10.75)	0.299
Diabetes duration, years	10.50 (16.50)	8.00 (6.50)	10.00 (1 1.00)	0.375
BMI, kg/m^2^	30.85 (4.72)	29.10 (5.95)	29.55 (8.05)	0.886
Waist circumference, cm	101.50 (8.12)	99.00 (8.00)	101.50 (15.00)	0.956
Handgrip strength, kg	22.00 (2.05)	23.40 (7.05)	24.45 (7.17)	0.362
SPPB score, 0-12	12.00 (0.25)	1 1.00 (1.50)	12.00 (1.00)	0.195
FFMI-DXA, kg/m^2^	18.32 (1.99)	17.36 (1.84)	17.35 (2.42)	0.690
Baumgartner index, kg/m^2^	7.94 (1.90)	7.76 (1.17)	7.49 (1.54)	0.730
FNIH index	0.66 (0.06)	0.61 (0.08)	0.59(0.08)	0.103
Laboratory variables				
HbA1c, %	7.70 (0.87)	8.20 (2.00)	7.55 (2.82)	0.683
Fasting glucose, mg/dL	105.00 (24.50)	130.00 (48.50)	132.50 (67.50)	0.459
eGFR (CKD-EPI), mL/min/1.73 m^2^	91.18 (15.94)	102.00 (10.73)	99.48 (21.52)	0.228
LDL cholesterol, mg/dL	98.10 (4.40)	102.00 (63.10)	98.30 (59.07)	0.956
HDL cholesterol, mg/dL	51.50 (9.25)	54.00 (19.50)	51.00 (21.25)	0.832
Triglycerides, mg/dL	132.00 (30.50)	143.00 (161.00)	134.50 (84.50)	0.954
Total cholesterol, mg/dL	180.00 (21.25)	200.00 (77.50)	182.50 (71.50)	0.859
Vitamin D, ng/mL	29.75 (5.35)	31.00 (13.34)	29.81 (9.10)	0.968
Calcium, mg/dL	9.50 (0.82)	9.60 (0.70)	9.50 (1.02)	0.541

Participants were categorized by serum P3NP (µg/L) as low (<14; n = 4), normal (14-31; n = 15), and high (>31; n = 80). Values are presented as median (interquartile range). BMI: Body mass index; CKD-EPI: chronic kidney disease epidemiology collaboration; DXA: dual-energy X-ray absorptiometry; eGFR: estimated glomerular filtration rate; FFMI-DXA: fat-free mass index by DXA; FNIH index: DXA-derived appendicular lean mass/BMI; HDL: high-density lipoprotein; LDL: low-density lipoprotein; P3NP: procollagen type III N-terminal propeptide; SPPB: Short Physical Performance Battery. *Kruskal-Wallis test.

Consistently, P3NP category distribution was similar across sarcopenia definitions, comorbidities, or functional strata **(Table 3).** For DXA-FFMI-defined sarcopenia, cases and non-cases exhibited similar proportions of low/normal/high P3NP: 3.0%/l4.9%/82.l% vs 6.3%/l5.6%/78.l% (Fisher's exact *p* = 0.744). Patterns were likewise non-significant for the Baumgartner index (*p* = l.000) and FNIH (*p* = 0.537). P3NP distributions did not differ significantly by postmenopausal status *(p* = 0.380), insulin use *(p* = 0.396), smoking *(p* = 0.8l3), alcohol use *(p* = 0.860), hypertension (*p* = l.000), peripheral neuropathy (*p* = 0.572), or prior COVID-l9 (*p* = 0.553), nor across IPAQ categories (*p* = 0.422) or SPPB strata (*p* = 0.269).

**Table 3:** Distribution of P3NP categories across sarcopenia definitions, comorbidities, and physical performance

**Variable**	**n**	**P3NP levels (µg/L)**			*p*-Value*
		**< 14**	**14-31**	**> 31**	
		**Low (n = 4)**	**Normal (n = 15)**	**High (n = 80)**	
Sarcopenia diagnosis					
Sarcopenia by FFMI-DXA					
Yes	67	2 (3.0)	10 (14.9)	55 (82.1)	0.744
No	32	2 (6.3)	5 (15.6)	25 (78.1)	
Sarcopenia by Baumgartner index					
Yes	2	0 (0.0)	0 (0.0)	2 (100)	1.000
No	97	4 (4.1)	15 (15.5)	78 (80.4)	
Sarcopenia by FNIH index					
Yes	8	0 (0.0)	0 (0.0)	8 (100)	0.537
No	91	4 (4.4)	15 (16.5)	72 (79.1)	
Comorbidities					
Postmenopausal					
Yes	82	3 (3.7)	14 (17.1)	65 (79.3)	0.380
No	17	1 (5.9)	1 (5.9)	15 (88.2)	
Insulin use					
Yes	36	1 (2.8)	3 (8.3)	32 (88.9)	0.396
No	63	3 (4.8)	12 (19.0)	48 (76.2)	
Smoker					
Yes	12	0 (0.0)	1 (8.3)	11 (91.7)	0.813
No	87	4 (4.6)	14 (16.1)	69 (79.3)	
Alcohol use					
Yes	16	0 (0.0)	3 (18.8)	13 (81.3)	0.860
No	83	4 (4.8)	12 (14.5)	67 (80.7)	
Hypertension					
Yes	71	3 (4.2)	11 (15.5)	57 (80.3)	1.000
No	28	1 (3.6)	4 (14.3)	23 (82.1)	
Peripheral neuropathy - feet					
Yes	38	2 (5.3)	4 (10.5)	32 (84.2)	0.572
No	61	2 (3.3)	11 (18.0)	48 (78.7)	
COVID-19 infection					
Yes	46	3 (6.5)	7 (15.2)	36 (78.3)	0.553
No	53	1 (1.9)	8 (15.1)	44 (83.0)	
Physical performance					
IPAQ					
Very active	15	0 (0.0)	2 (13.3)	13 (86.7)	0.422
Active	40	3 (7.5)	6 (15.0)	31 (77.5)	
Irregularly active A	5	1 (20.0)	1 (20.0)	3 (60.0)	
Irregularly active B	35	0 (0.0)	5 (14.3)	30 (85.7)	
Sedentary	4	0 (0.0)	1 (25.0)	3 (75.0)	
SPPB					
Autonomous	89	4 (4.5)	13 (14.6)	72 (80.9)	0.269
Pre-Frail	9	0 (0.0)	1 (11.1)	8 (88.9)	
Frail	1	0 (0.0)	1 (100)	0 (0.0)	

Participants were classified by serum P3NP as low (<14 µg/L; n = 4), normal (14-31 µg/L; n = 15), and high (>31 µg/L; n = 80). Values are presented as n (%). COVID-19: coronavirus disease 2019; DXA: dual-energy X-ray absorptiometry; FFMI: fat-free mass index; FNIH index: DXA-derived appendicular lean mass/BMI; IPAQ: International Physical Activity Questionnaire; P3NP: procollagen type III N-terminal propeptide; SPPB: Short Physical Performance Battery. *Fisher's exact test

In the overall sample, serum P3NP showed a weak inverse correlation with age (Spearman's r = -0.210, *p* = 0.037; **Figure 1A)** and a weak positive correlation with SPPB (r = 0.225, *p* = 0.025; **Figure 1B).** No additional correlations with P3NP were detected. Given that only two correlations were prespecified, Holm adjustment yielded p_holm = 0.050 for both; these effects should be interpreted as small and borderline in terms of significance.

**Figure 1:**
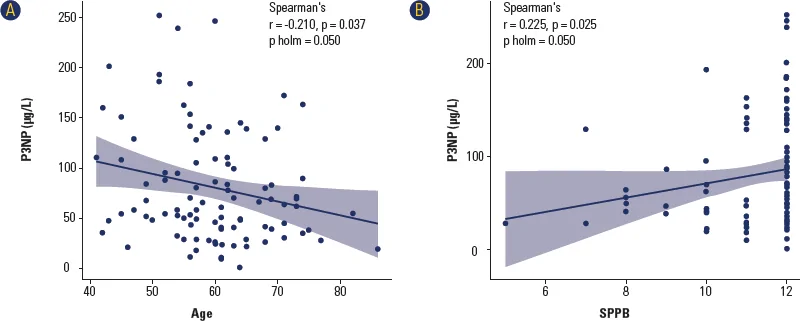
Serum P3NP scatterplots with LOESS smoothing. **A)** P3NP (µg/L) versus age (years); points denote individual participants and the solid line depicts the LOESS fit. Spearman's *p* = -0.210, *p* = 0.037; Holm-adjusted *p* = 0.050 (two comparisons). **B)** P3NP (µg/L) versus SPPB score (0-12); Spearman's *p* = 0.225, *p* = 0.025; Holm-adjusted *p* = 0.050. Correlations are two-tailed; Shaded bands denote 95% confidence intervals for the LOESS fits.

LOESS: locally estimated scatterplot smoothing; P3NP: procollagen type III N-terminal propeptide; SPPB: Short Physical Performance Battery.

As a single marker, P3NP showed limited discrimination for sarcopenia: AUC 0.532 (FFMI-DXA outcome). In a prespecified clinical model with age, BMI, HbA1c, eGFR ("clinical model"), adding log-P3NP (z-score per 1 SD) did not improve discrimination: AUC 0.896 (clinical) vs 0.902 (clinical + P3NP); ΔAUC = +0.0069 (bootstrap 95% CI -0.0046 to +0.0332). P3NP was not independently associated with sarcopenia (OR per 1 SD 1.37, 95% CI 0.84-2.23; *p* = 0.211), while BMI retained a significant inverse association (OR 0.68, 95% CI 0.53-0.89; *p* = 0.004). Model calibration was adequate (Hosmer-Lemeshow *p* = 0.18).

Including an interaction term revealed effect modification: the interaction between log-transformed, z-standardized P3NP and BMI was significant for DXA-FFMI-defined sarcopenia (interaction OR 0.84, 95% CI 0.75-0.95; *p* = 0.005), indicating that the association between P3NP and sarcopenia was stronger at lower BMI. Within this interaction model, each 1-SD increase in log-P3NP was independently associated with sarcopenia (OR 1.65, 95% CI 1.01-2.68; *p* = 0.044), with high model discrimination (AUC 0.909). Predicted probabilities by P3NP (z-score) for BMI quartiles and WHO BMI categories are adjusted for other covariates held at sample means **(Figure 2B).**

**Figure 2:**
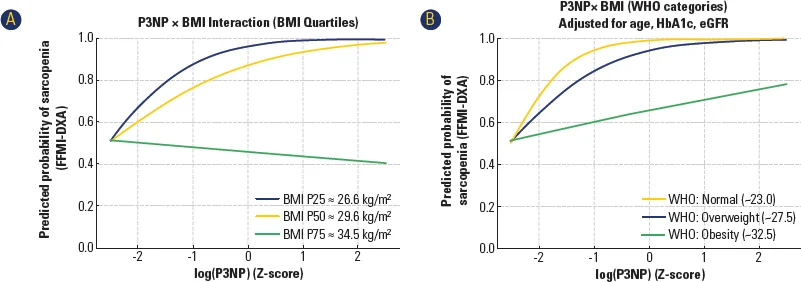
P3NP × BMI interaction for FFMI-DXA-defined sarcopenia. A) Predicted probability of sarcopenia across log-transformed, z-standardized P3NP (x-axis) at BMI quartiles (P25 ≈ 26.6, P50 ≈ 29.6, P75 ≈ 34.5 kg/m^2^). B) Predicted probabilities at representative WHO BMI categories (normal weight ≈ 23.0 kg/m^2^, overweight ≈ 27.5 kg/m^2^, obesity ≈ 32.5 kg/m^2^). Curves are derived from a multivariable logistic regression including the P3NP × BMI interaction, adjusted for age, HbA1c, and eGFR; all covariates are held at their sample means (BMI centered at the sample mean). The interaction indicates a stronger positive association of P3NP with sarcopenia at lower BMI (interaction OR 0.84, 95% CI 0.75-0.95; *p =* 0.005); model AUC ≈ 0.909 (n ≈ 99).

AUC: area under the ROC curve; BMI: body mass index; CI: confidence interval; DXA: dual-energy X-ray absorptiometry; eGFR: estimated glomerular filtration rate; FFMI: fat-free mass index; HbA1c: glycated hemoglobin; OR: odds ratio; P3NP: procollagen type III N-terminal propeptide; WHO: World Health Organization.

Exploration of effect modification by glycemic status (HbA1c) did not substantially alter these conclusions. In a model with HbA1c dichotomized (<7.4% vs ≥7.4%), the P3NP × HbA1c interaction was not statistically significant (interaction OR 0.29, 95% CI 0.06-1.28; *p* = 0.103). Within strata, P3NP (log-z) had a borderline positive association in the high HbA1c group (≥7.4%) (OR 1.81, 95% CI 0.97-3.38; *p* = 0.062) and a non-significant inverse association in the low HbA1c group (<7.4%) (OR 0.57, 95% CI 0.18-1.77; *p* = 0.328). Model discrimination remained high (AUC 0.917), though calibration was suboptimal overall (Hosmer-Lemeshow *p* = 0.0007), acceptable in the high HbA1c stratum (HL *p* = 0.4415), and poor in the low HbA1c stratum (HL *p* = 0.0002) **(Figure 3A** and **Supplementary Table S1).** Model calibration metrics and relevant *p*-values are provided in **Supplementary Table S1.**

**Figure 3:**
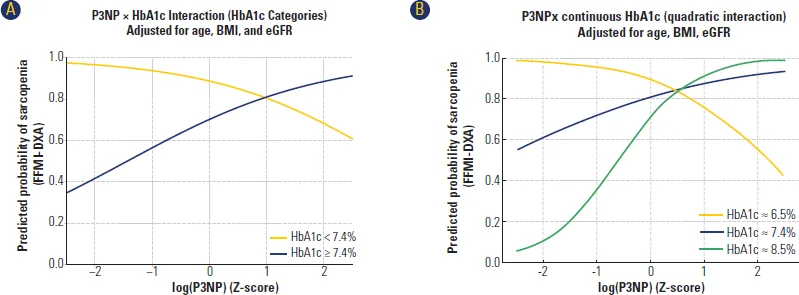
Glycemic status and the association between P3NP and DXA-FFMI sarcopenia. **A)** Predicted probability of DXA-FFMI-defined sarcopenia across log-transformed, z-standardized P3NP (x-axis), stratified by HbA1c category (<7.4% vs ≥7.4%). Estimates are from a multivariable logistic model including age (z), BMI (z), eGFR (z), log-P3NP (z), HbA1c category, and the log-P3NP×HbA1c interaction; all continuous covariates are held at their sample means. **B)** Predicted probability of DXA-FFMI sarcopenia across log-P3NP (z) with HbA1c modeled as a continuous variable (quadratic polynomial) to illustrate the shape of the P3NP-glycemia relationship; curves are shown at representative HbA1c values (e.g., lower, median, and upper sample percentiles), with other covariates fixed at sample means. Interaction results provided in **Supplementary Tables S1-S2;** these visualizations do not alter the primary conclusion that P3NP offers limited independent discrimination.

BMI: body mass index; CI: confidence interval; DXA: dual-energy X-ray absorptiometry; eGFR: estimated glomerular filtration rate; FFMI: fat-free mass index; HbA1c: glycated hemoglobin; P3NP: procollagen type III N-terminal propeptide.

Modeling HbA1c as a continuous variable using a quadratic polynomial, with interactions between P3NP and both the linear and quadratic HbA1c terms, did not provide statistically significant evidence of effect modification: the likelihood-ratio test for the interaction yielded χ^2^ = 4.87, df = 2, *p* = 0.0876, and the joint Wald test yielded χ^2^ = 4.75, df = 2, *p* = 0.0929. Predicted probability curves at HbA1c values of 6.5%, 7.4%, and 8.5% revealed only minor divergence across glycemic levels **(Figure 3** and **Supplementary Table S2).**

Five-fold cross-validation of the HbA1c interaction model provided a mean AUC of 0.898 (SD 0.061) overall; within the high HbA1c stratum (≥7.4%), the AUC was 0.869 (SD 0.088; n = 56, 37 events), whereas within the low HbA1c stratum (<7.4%), the AUC was 0.907 (SD 0.146; n = 43, 30 events) **(Supplementary Table S3).**

Collectively, analyses incorporating HbA1c did not alter the main conclusion that P3NP lacks independent clinical utility for DXA-FFMI-defined sarcopenia in this study. Excluding participants with eGFR< 60 mL/min/1.73 m^2^ did not affect conclusions: P3NP OR per 1 SD 1.42 (95% CI 0.87-2.31; *p =* 0.163), AUC 0.899. In multiple linear regression with log-P3NP as the dependent variable (age, BMI, HbA1c, eGFR, handgrip, SPPB, physical activity, menopause), no predictor showed independent association (all *p* > 0.16; adjusted R^2^ ≈ 0.00).

## DISCUSSION

DXA-Derived FFMI-defined sarcopenia was common, associated with lower BMI, waist circumference, FFMI, and the Baumgartner index, and modestly lower handgrip strength, but SPPB performance was similar between women with and without sarcopenia. Circulating P3NP showed only weak associations, including a small inverse correlation with age and a small positive correlation with SPPB, and did not discriminate clinical, functional, or biochemical features across P3NP strata. Taken together, these data indicate that in women with T2DM, P3NP alone is not an informative standalone biomarker for sarcopenia or related performance phenotypes. Consistent with this interpretation, adding P3NP to a prespecified clinical model (age, BMI, HbA1c, eGFR) yielded a minimal, non-meaningful gain in discrimination (ΔAUC ≈ 0.007), reinforcing the limited incremental utility of P3NP in this context.

In our study, 80.8% of participants had elevated P3NP, which plausibly reflects extra-muscular fibrogenesis, particularly hepatic, a common comorbidity in T2DM (^29^,^30^). This high prevalence of elevated P3NP may have reduced between-group variability and limited its discriminatory ability, potentially creating a ceiling effect. In populations where biomarker distributions are markedly elevated, statistical contrasts between categories are constrained, explaining the limited performance of P3NP observed.

A French biopsy-based study of 330 individuals with T2DM found 38% had advanced fibrosis (F3-F4), even with low alanine aminotransferase thresholds (^29^). Recent consensus statements estimate that ~20% of individuals with T2DM have significant fibrosis and over 70% have metabolic dysfunction-associated steatotic liver disease, formerly referred to as nonalcoholic fatty liver disease (^30^). Moreover, P3NP may capture adipose-tissue fibro-inflammation in T2DM, further diluting hepatic/muscle specificity when used in isolation (^31^). Collectively, this context explains both the widespread elevation of P3NP and its weak standalone predictive value for sarcopenia. Accordingly, P3NP is better positioned as part of multimarker panels (e.g., ELF), interpreted alongside clinical, functional, and metabolic variables (^30^,^32^).

### Comparison with prior research

Several population-based studies have associated higher P3NP with poorer physical function or muscle status, although results have been inconsistent and context-dependent. In the Long Life Family Study, Santanasto and cols. (^13^) reported that higher plasma P3NP related to worse baseline physical function (lower SPPB, slower gait, weaker grip) and greater seven-year decline (all *p* < 0.001). In older Korean men, Shin and cols. (^17^) found higher P3NP with lower physical performance. In the InCHIANTI study, Pellegrino and cols. (^16^) observed that circulating P3NP predicted poorer muscle quality and performance. Contrarily, Chen and cols. (^18^) in Taiwanese elders found apelin and HtrA1, but not P3NP, were more consistently associated with sarcopenia status; P3NP showed weak or null associations after adjustment and only modest ROC performance (AUROC ≈ 0.58) (^19^). Our results more closely align with the latter: P3NP showed only small correlations and no convincing cross-sectional differences across clinical or functional strata, reinforcing that single-marker performance is limited and study-specific. Consistent with prior findings, the small ΔAUC observed and strong performance of clinical covariates alone indicate that P3NP adds little incremental value beyond established risk factors in women with T2DM (^13^,^16^,^17^,^22^).

### Potential mechanisms for P3NP underperformance in women with T2DM

P3NP reflects systemic type III collagen turnover, not exclusively skeletal muscle, integrating multiple remodeling processes (^13^). Collagen III has tissue- and context-dependent roles in compliance and repair; its signaling is intricately linked to fibroblast-matrix mechanisms (^33^) and is co-regulated with collagen I during tissue injury and regeneration (^15^). In T2DM, systemic inflammation and pyroptosis can drive maladaptive remodeling and muscle loss through non-myocentric pathways (^34^), while antihyperglycemic therapies themselves may differentially influence muscle mass/function (^35^), all of which could dilute any direct myofibrotic "signal" captured by P3NP. Moreover, P3NP exhibits age-, sex-, and physiology-dependent distributions; Northern European reference intervals differ by age and sex and are influenced by cardiometabolic covariates (^22^). These features, together with the high cardiometabolic burden in T2DM, may explain for the weak, nonspecific correlations we observed. Notably, our HbA1c analyses yielded mixed signals (likelihood-ratio positive; joint Wald negative) and poorer calibration in the low-HbA1c stratum, suggesting any glycemia-related modulation of the P3NP-sarcopenia relationship is small and statistically unstable.

### Position within the biomarker landscape

Consensus reviews have emphasized that sarcopenia is multifactorial and unlikely to be captured by a single circulating biomarker (^2^,^3^). Contemporary recommendations advise that blood-based markers should complement, not replace, direct measures of strength, mass, or performance (^11^). Recent work advocates for combining markers spanning inflammation, extracellular matrix turnover, metabolism, and neuromuscular status (^23^,^24^). Our null-leaning P3NP findings in women with T2DM are concordant with the field's shift from single-to multi-analyte strategies. Accordingly, P3NP is better positioned as one component within extracellular matrix-oriented panels rather than as a stand-alone screening biomarker in this population (^23^,^24^).

### Clinical and methodological implications

For diabetic sarcopenia, where metabolic disease shapes muscle biology, biomarker interpretation should explicitly consider diabetes status and its management (^7^,^36^). Our results support current guidelines prioritizing standardized phenotyping (DXA/ALM, grip strength, SPPB) while considering biomarkers as adjuncts rather than diagnostic gatekeepers (^11^,^12^). Given assay heterogeneity and broad physiologic determinants of P3NP, interpretation should be grounded in population-appropriate reference intervals and to clearly defined clinical question (screening, prognosis, or pharmacodynamics) (^22^). We therefore do not recommend P3NP for stand-alone screening of DXA-defined sarcopenia in women with T2DM. When reported, calibration (Hosmer-Lemeshow, intercept/slope, Brier score) and cross-validated discrimination should accompany ΔAUC when assessing clinical utility (^11^,^12^).

### Strengths and limitations

This study's strengths include rigorous, DXA-based body composition criteria, comprehensive phenotyp-ing, and prespecified P3NP hypotheses. Limitations include the primarily cross-sectional design; potential residual confounding by medication class, glycemic control, or comorbidities; and small numbers in the low-P3NP group, limiting power for threshold-based analyses. More broadly, P3NP's systemic origin and broad extracellular matrix involvement may weaken its specificity for muscle-related changes in metabolically complex populations, as echoed in prior reviews (^2^). Additionally, our primary definition relied on FFMI with BMI-specific thresholds to account for adiposity, potentially limiting comparability to studies using alternative indices or fixed cut-points. Although overall events-per-variable were adequate, stratified HbA1c models approached EPV <10 in small cells (e.g., P3NP <14 µg/L), raising concerns regarding precision and calibration. In addition, although the clinical model showed high apparent discrimination, internal validation procedures (bootstrap optimism correction and cross-validation) were applied to mitigate potential optimism bias and to provide a more conservative estimate of model performance.

### Future directions

Our findings suggest multimarker panels integrating extracellular matrix remodeling (e.g., P3NP), inflammatory, metabolic, and neuromuscular indices, ideally paired with longitudinal functional trajectories and intervention responsiveness (^13^,^23^,^24^). Stratified analyses by menopausal status and diabetes treatment are warranted to clarify effect modification in women, an area repeatedly emphasized as clinically important in aging/sarcopenia roadmaps (^11^,^12^), and should reference population-specific intervals (^22^). Lastly, prospective studies in T2DM that track P3NP alongside DXA/ALM, strength, and performance, before and after targeted lifestyle or pharmacologic interventions, will be critical to establish whether P3NP contributes independent, actionable information beyond goldstandard phenotypes. Future studies should prioritize external validation, prespecified continuous HbA1c interactions with adequate power for effect modification, and inclusion of decision-curve analysis and calibration metrics alongside ΔAUC to judge clinical usefulness.

In conclusion, in this single-center, cross-sectional study of women with T2DM, DXA-derived FFMI-defined sarcopenia was associated with marked differences in body composition but only modest differences in handgrip strength and largely comparable laboratory profiles. Circulating P3NP was only weakly associated with clinical and functional measures and did not meaningfully enhance discrimination beyond a prespecified clinical model (age, BMI, HbA1c, eGFR; ΔAUC ≈ 0.007). Exploratory effect modification analyses (P3NP × BMI; P3NP × HbA1c, categorical and continuous), cross-validated performance, and calibration assessment did not alter this conclusion. P3NP levels were elevated in 80.8% of participants, a finding that may reflect the high background prevalence of hepatic fibrosis and metabolic dysfunction-associated steatotic liver disease, as well as contributions from extra-hepatic fibro-inflammatory processes. Overall, these findings do not support the use of P3NP as a standalone bio-marker for sarcopenia in women with T2DM and suggest that it should instead be interpreted within the context of multimarker panels and clinical-metabolic characteristics. P3NP is therefore not recommended for stand-alone screening of DXA-defined sarcopenia in women with T2DM. Standardized phenotyping incorporating muscle quantity (DXA), strength, and performance remains essential; P3NP may, at most, serve as an adjunct indicator of extracellular matrix turnover. Given the single-center, cross-sectional design, both generalizability and causal inference are limited.

## Figures and Tables

**Supplementary Figure S1:**
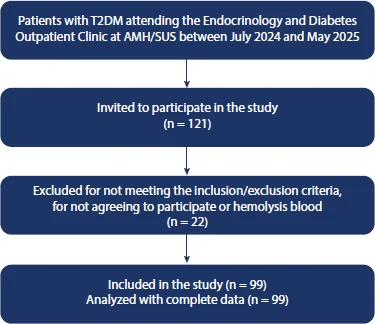
Study flow. Of 121 individuals assessed, 22 were excluded (ineligible, n = 9; declined, n = 7; hemolyzed samples, n = 6), yielding 99 participants included in the final analysis.

AMH: Agamenon Magalhães Hospital; SUS: Brazilian Unified Health System.

**Supplementary Table S1A:** Logistic regression coefficients for DXA-FFMI sarcopenia with HbA1c category and P3NP×HbA1c interaction

**Variable**	**Estimate**	**SE**	**Odds Ratio**	**Lower 95%**	**Upper 95%**	***p*-Value**
Intercept	2.072	0.655	7.941	2.200	28.656	0.0016
log_P3NP_z	-0.649	0.628	0.523	0.153	1.790	0.3016
HbA1c_category	-1.214	0.747	0.297	0.069	1.285	0.1042
log_P3NP_z:HbA1c_category	1.243	0.747	3.465	0.801	14.992	0.0964
Age_z	0.189	0.395	1.209	0.557	2.621	0.6316
BMI_z	-2.464	0.548	0.085	0.029	0.249	0.0000
eGFR_z	-0.079	0.360	0.924	0.456	1.872	0.8262

Logistic model for DXA-FFMI-defined sarcopenia (event=1) including age (z), BMI (z), eGFR (z), log-P3NP (z), HbA1c category (<7.4% vs ≥7.4%), and the P3NP×HbA1c interaction. The table reports log-odds estimates, standard errors, odds ratios (exp(β)), 95% confidence intervals, and p-Values. BMI: body mass index; eGFR: estimated glomerular filtration rate; HbA1c: glycated hemoglobin; P3NP: procollagen type III N-terminal propeptide; SE: standard errors; z: z-standardized.

**Supplementary Table S1B:** Discrimination and calibration for the HbA1c-category interaction model, overall and by HbA1c stratum

**Measure**	**Value**	**95% CI**
AUC (overall)	0.917	(0.837, 0.979)
Hosmer-Lemeshow p (overall)	0.0007	
AUC (low HbA1c (<7.4%))	0.923	(0.750, 1.000)
Hosmer-Lemeshow p (Low HbA1c (<7.4%))	0.0002	
AUC (high HbA1c (≥7.4%))	0.890	(0.786, 0.973)
Hosmer-Lemeshow p (High HbA1c (≥7.4%))	0.4415	

Model performance for the specification in S1A. Area under the ROC curve (AUC) with bootstrap 95% CI and Hosmer-Lemeshow (HL) goodness-of-fit p-Values are shown overall and separately for Low HbA1c (<7.4%) and High HbA1c (≥7.4%) strata using the model's predicted probabilities. Note: HL p-Values in small strata should be interpreted with caution. AUC: area under the ROC curve; CI: confidence interval; HbA1c: glycated hemoglobin; HL: Hosmer-Lemeshow; p: p-valor; ROC: receiver operating characteristic.

**Supplementary Table S2A:** Coefficients for the model with continuous HbA1c (quadratic polynomial) and P3NP×HbA1c interaction

**Variable**	**Estimate**	**SE**	**Odds Ratio**	**Lower 95%**	**Upper 95%**	***p*-Value**
Intercept	11.507	5.095	99432.903	4.574	2161333819.562	0.0239
log_P3NP_z	-9.505	4.516	0.000	0.000	0.520	0.0353
Age_z	0.172	0.420	1.188	0.522	2.704	0.6822
BMI_z	-2.588	0.585	0.075	0.024	0.237	0.0000
eGFR_z	-0.153	0.405	0.858	0.388	1.897	0.7059
HbA1c	-2.195	1.194	0.111	0.011	1.157	0.0661
HbA1c_sq	0.109	0.070	1.115	0.973	1.278	0.1182
log_P3NP_z:HbA1c	2.312	1.089	10.096	1.195	85.320	0.0337
log_P3NP_z:HbA1c_sq	-0.131	0.067	0.877	0.769	1.000	0.0504

Logistic model for DXA-FFMI-defined sarcopenia with age (z), BMI (z), eGFR (z), log-P3NP (z), and HbA1c modeled as a continuous variable using quadratic polynomial; interaction terms between log-P3NP (z) and spline(HbA1c) are included. The table reports log-odds estimates, standard errors, odds ratios (exp(β)), 95% confidence intervals, and p-Values. The quadratic polynomial used is indicated in S2B. BMI: body mass index; CI: confidence interval; DXA: dual-energy X-ray absorptiometry; eGFR: estimated glomerular filtration rate; FFMI: fat-free mass index; HbA1c: glycated hemoglobin; P3NP: procollagen type III N-terminal propeptide; SE: standard errors; sq: squared (quadratic term); z: z-standardized.

**Supplementary Table S2B:** Interaction tests for P3NP×HbA1c (continuous): likelihood-ratio and Wald joint tests

**Test**	**Chi-square**	**df**	***p*-Value**
Likelihood-ratio (interaction terms)	4.87	2	0.0876
Wald (joint, interaction terms)	4.75	2	0.0929
Basis used	poly2		

The likelihood-ratio (LR) test compares the spline model with versus without the P3NP×HbA1c interaction. The Wald test evaluates the joint null that all interaction coefficients equal zero. The final row indicates the basis used for HbA1c (quadratic polynomial). df: degrees of freedom; HbA1c: glycated hemoglobin; P3NP: procollagen type III N-terminal propeptide; Wald: Wald chi-square test.

**Supplementary Table S3A:** Five-fold cross-validated AUCs by fold (overall and by HbA1c stratum) for the HbA1c-category interaction model

**Fold**	**AUC (overall)**	**AUC (High HbA1c)**	**AUC (Low HbA1c)**
1	0.798	0.889	0.667
2	0.917	0.861	1.000
3	0.912	0.833	1.000
4	0.901	0.760	1.000
5	0.962	1.000	0.867

Stratified 5-fold cross-validation of the logistic model with log-P3NP (z), age (z), BMI (z), eGFR (z), HbA1c category (<7.4% vs ≥7.4%), and the P3NP×HbA1c interaction. Z-scores are computed within each training fold; predictions and AUCs are evaluated on the held-out fold. Per-fold AUCs are shown for the full test set (overall) and separately for High HbA1c (≥7.4%) and Low HbA1c (<7.4%) subsets. AUC: area under the ROC curve; BMI: body mass index; eGFR: estimated glomerular filtration rate; HbA1c: glycated hemoglobin; ROC: receiver operating characteristic; z: z-standardized.

**Supplementary Table S3B:** Cross-validated AUC (mean ± SD) overall and by HbA1c stratum

**Fold**	**AUC (overall)**	**AUC (High HbA1c)**	**AUC (Low HbA1c)**
Mean ± SD	0.898 ± 0.061	0.869 ± 0.088	0.907 ± 0.146

Summary of the 5-fold cross-validation in S3A, presenting the mean ± SD of AUCs overall, in High HbA1c (≥7.4%), and in Low HbA1c (<7.4%) strata. Values reflect performance on held-out folds. AUC: area under the ROC curve; HbA1c: glycated hemoglobin; SD: standard deviation.

## SUPPLEMENTARY MATERIALS

**Supplementary Table S1.** Logistic regression with HbA1c category (<7.4% vs ≥7.4%)

**Model coefficients**

**Model performance**

**Supplementary Table S2.** HbA1c modeled as continuous (quadratic polynomial) with P3NP interaction

**Model coefficients**

**Interaction tests**

**Supplementary Table S3.** 5-fold cross-validated AUCs (interaction model with HbA1c category)

**Per-fold AUCs**

**Summary**
